# 1524. Racial and Gender Disparities in HIV Mortality: A population-based Retrospective Study in the United States from 1999 to 2020

**DOI:** 10.1093/ofid/ofad500.1359

**Published:** 2023-11-27

**Authors:** Monique A Prince, Min-Choon Tan, Min-Xuan Tan, E’ebony Prince, Rick Nicholas, Hamid Shaaban, Jihad Slim

**Affiliations:** Saint Michael's Medical Center, Newark, New Jersey; Saint Michael's Medical Center, Newark, New Jersey; Monash University, Melbourne, Victoria, Australia; St. George's University, True Blue, Saint George, Grenada; University of Technology, 237 Old Hope Road, Kingston, Jamaica; St. Michael Medical Center, Newark, New Jersey; Saint Michael’s Medical Center, Newark, NJ, USA, Newark, New Jersey

## Abstract

**Background:**

Human Immunodeficiency Virus (HIV) has been historically associated with high mortality however, rates have declined with expanded access to antiretroviral therapy. It is important to identify and dissect inequities that contribute to mortality rates in individuals with HIV, which may help inform targeted interventions to improve treatment access in future decades. This study sought to determine the longitudinal trends in mortality attributed to HIV disease by race and sex.

**Methods:**

We queried the Centers for Disease Control and Prevention's Wide-Ranging Online Data for Epidemiologic Research (CDC WONDER) database and performed serial cross-sectional analyses of national death certificate data for HIV-related mortality. HIV diseases (ICD-10 B20-B24, O98.7, R75) were listed as the underlying cause of death. We calculated age-adjusted mortality rates (AAMR) per 1,000,000 individuals and determined the trends over time by using the Joinpoint Regression Program. Subgroup analyses were performed by sex and race.

**Figure 1:**
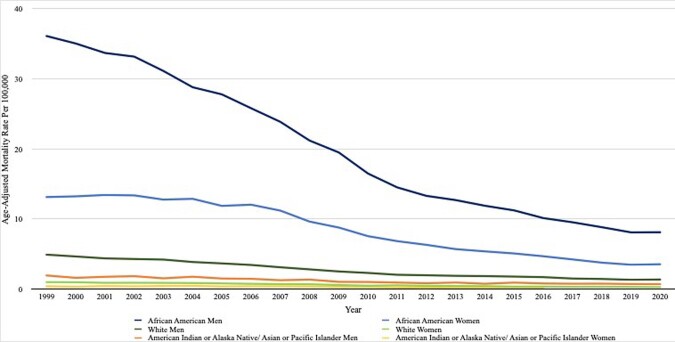
Demonstrates Age-Adjusted HIV Mortality Rate per 100,000

**Results:**

In the 22-year study period, 210 921 HIV-related mortality in the United States were identified between 1999 and 2020. The AAMR from HIV-related mortality decreased significantly from 5.32 per 1,000,000 individuals in 1999 to 1.40 per 1,000,000 individuals in 2020 (p< 0.01). Overall, men recorded a higher AAMR compared to women (4.66 vs. 1.65 per 1,000,000 individuals, p< 0.01). African American individuals had the highest AAMRs (13.46 per 1,000,000 individuals) compared to White, American Indian, and Asian individuals (1.70 vs 1.65 vs 0.47 per 1,000,000 individuals). Similar decreasing trends were seen in all racial groups: African American men from 36.12 to 8.11 per 1,000,000 individuals, African American women from 13.13 to 3.54 per 1,000,000 individuals, White men from 4.91 to 1.36 per 1,000,000 individuals, and White women from 1.00 to 0.27 per 1,000,000 individuals.

**Conclusion:**

The AAMR from HIV-related mortality has decreased significantly over the last two decades with significant racial and gender disparities. Men and African American individuals have higher AAMR from HIV-related mortality. This highlights the effectiveness of antiretroviral medications and emphasizes the need to improve the healthcare access among those underserved population.

**Disclosures:**

**Jihad Slim, MD, FACP**, ViiV Healthcare: Advisor/Consultant|ViiV Healthcare: Grant/Research Support

